# Evaluation of CTX-I, CTX-II, TIMP-I, MMP-9 and PIICP in the population of working German Shepherd dogs in Slovakia

**DOI:** 10.17221/127/2023-VETMED

**Published:** 2024-10-30

**Authors:** Scarlett Maresova, Tomas Liptak, Aladar Madari, Patrik Zeleznik, Csilla Tothova, Maria Kuricova

**Affiliations:** Small Animal Cinic, University of Veterinary Medicine and Pharmacy in Košice, Košice, Slovak Republic

**Keywords:** blood, cartilage, C-terminal telopeptides, metalloproteinase, urine

## Abstract

The aim of this study was to determine the reference values of cartilage damage biomarkers in the blood and urine in 76 clinically healthy German Shepherd dogs. We grouped the dogs into 4 groups by age. All the groups were consistently made up of 19 dogs. The mean age and average body weight were established for all the dog groups. The blood was collected from the *vena cephalica antebrachii* or the *vena saphena medialis* and the urine sample was taken by us-guided cystocenthesis. The biomarkers were determined using quantitative sandwich ELISA kits. The mean values of the biomarkers ± SD were, for the urine biomarkers, CTX-I 3.29 ± 1.16 ng/ml, CTX-II 1 993.95 ± 777.04 ng/ml, and TIMP-I 392.80 ± 160.56 ng/ml, and for the blood biomarkers, MMP-9 89.85 ± 50.21 ng/ml and PIICP 19.19 ± 7.33 ng/ml. Based on the obtained values of the mean 95%, we expect a standard for CTX-I 5.05 ng/ml, CTX-II 3 204.26 ng/ml, TIMP-I 606.64 ng/ml, MMP-9 187.93 ng/ml, and PIICP 31.71 ng/ml.

There is a growing amount of data in the literature on the association of blood, urine, or synovial biomarker values in a variety of diseases in both dogs and humans. Many of the studies have provided indications that these markers could not be specific for one particular disease, as their changing levels, increasing or decreasing, have been recorded in several disease processes. There is also an indication that there may be some age-related diversity with regards to the biomarkers.

The developmental and homeostatic remodelling of the extracellular matrix (ECM) is a highly regulated process orchestrated by a family of zinc-containing, calcium-dependent, secreted neutral proteases known as the matrix metalloproteinases (MMPs). This family of enzymes, which now contains over 25 members, can collectively degrade all structural proteins of the ECM, including interstitial collagens (I, II, III and V), basement membrane collagen (IV), fibronectin, laminin, proteoglycans and elastin. The MMPs include collagenases, gelatinases, stromelysins, macrophage metalloelastase, matrilysin, and membrane-type MMPs. They are regulated at the level of gene transcription by latent proenzyme activation and are inhibited by a group of inhibitor proteins known as tissue inhibitors of MMP, which consist of four family members (tissue inhibitor of metalloproteinases TIMP-1 to TIMP-4) (Birkedal-Hansen et al. 1993; Leco et al. 1997; Bonnans et al. 2014).

A highly regulated balance of active MMPs and TIMPs is maintained during normal tissue metabolism. The relative balance of MMPs and TIMPs is thought to determine the rate of the extracellular matrix (ECM) turnover. An imbalance in these ratios can contribute to the progression of some pathological disorders, including tumour invasion and metastasis, arthritis, fibrotic diseases, atherosclerosis, and emphysema (Ahner et al. 2019).

A study by Ahner et al. (2019) was the first to demonstrate statistically significant differences in protein biomarkers related to bone or cartilage repair in dogs with and without signs of canine hip dysplasia (CHD). A panel consisting of four biomarkers (urinary C-telopeptide fragments of type I collagen CTX-I and type II collagen, serum MMP-9 and Procollagen II C-Terminal Propeptide PIICP) was determined to be highly discriminatory in detecting the presence or absence of hip dysplasia in adult dogs. There is limited knowledge about the physiological values of cartilage damage biomarkers in veterinary medicine. However, there is some evidence of their use in pathological processes (Ahner et al. 2019).

Dogs with radiographic manifestations of CHD had a significantly lower concentration of CTX-II (a biomarker of the extracellular matrix breakdown) in the urine compared to dogs with radiographically normal hips. These findings suggest an altered metabolism of the extracellular matrix, which plays a role in the initiation and progression of osteoarthritis (OA).

CTX-II levels in the urine of dysplastic dogs were significantly lower compared to non-dysplastic dogs. A study by Ahner et al. (2019) showed no significant differences in the urinary or serum MMP-3 levels, but serum MMP-9 was one of the biomarkers that could distinguish adult dogs with hip dysplasia from dogs without it (Ahner et al. 2019).

Urinary CTX-II is considered a biomarker specific for cartilage breakdown, but not specific for hip joints (Nepple et al. 2015).

Dearmin et al. (2014) detected a biochemical change in dogs with a significant increase in the PIICP concentration in the serum as a result of the increased MMP enzyme activity (Dearmin et al. 2014).

C-terminal propeptides direct the intracellular assembly of procollagen molecules, leading to fibril formation (Hulmes 2019). Pathological processes in articular cartilage lead to the increased cleavage of type II collagen, which is accompanied by increased collagen synthesis, whereby PIICP directly reflects the rate of collagen synthesis (Nelson et al. 1998).

The purpose of this study was to evaluate the C-telopeptide of crosslinked collagen type I and type II (CTX-I, CTX-II) and metallopeptidase inhibitor 1 (TIMP-I) in urine samples, and the matrix metalloproteinase 9 (MMP-9), C-terminal propeptide of collagen type-II (PIICP) in venous blood samples of a cohort group of working German Shepherd dogs.

We evaluated the influence of the age and sex on the serum or urine levels of these biomarkers, which are studied as part of the diagnosis of various diseases including inflammatory, neoplastic or degenerative diseases, but most of the importance is given as an early predictive tool for osteoarthritis.

Since there can be a great variability in individual biomarkers, both the individual and age-related, as well as fluctuations in the levels of some biomarkers during the day have been described, our goal was to investigate a cohort of dogs of the same breed, of both sexes and various ages, and establish the levels that could serve as reference values for these working dogs.

## MATERIAL AND METHODS

This study included 76 clinically healthy dogs used in the services of the police force of both genders from the age of 2 months old to 144 months old, weighing from 13.9 to 42.7 kg, without clinical or past evidence of orthopaedic or systemic diseases. The group of dogs was divided into four groups: the first group from 2 to 12 months old, the second group from 13 to 36 months old, the third group from 37 to 60 months old and the fourth group of dogs older than 60 months.

We measured the body temperature, respiratory and heart rate and femoral pulse rate of the dogs under study before sampling. The dogs were then weighed and examined systematically (orthopaedic examination, neurological examination, X-ray, blood test for haematological and biochemical parameters). Following the collection of the venous blood from the *vena cephalica antebrachii* or the *vena saphena medialis*, the urine sample was taken by us-guided cystocenthesis. The samples were transported to the laboratory in a portable cooler where the temperature was kept at 7** °**C until arrival. We used the following commercially available canine-specific sandwich-type enzyme-linked immunosorbent assay (ELISA) tests from MyBioSource Inc. (San Diego, CA, USA): Canine Neurofilament-Light Chain (NFL) ELISA Kit (Cat. No. MBS9399591), Canine Tau Protein (TAU) ELISA Kit (Cat. No. MBS734093), Canine Amyloid Beta Peptide 1-42 ELISA Kit (Cat. No. MBS089848). We followed the manufacturer’s instructions. These non-validated kits contained a set of standards for the preparation of the calibration curve, but the control material was not available. We read the optical density at 450 nm using an Opsys MR microplate photometer reader from Dynex Technologies (Chantilly, VA, USA) and the results (levels of evaluated biomarkers) were then recalculated by the professional curve fitting software Revelation QuickLink v4.25 (Dynex Technologies, Chantilly, VA, USA).

All the gained data was statistically analysed using a one-way analysis of variance (ANOVA) test. Males and females were compared with a paired *t*-test. The calculation method for the reference interval with respect to the sample size, its descriptive statistics and the normality of the distribution was parametric with a logarithmic transformation. We evaluated all the variables using Pearson’s correlation analysis at *P* < 0.05.

## RESULTS

The mean age of the studied dogs, mean body weight, mean ± SD of the studied markers, upper 95% (percentile) in each group as well as the mean biomarker values and upper 95% for each weight group are listed in Table 1, Table 2 and Table 3.

**Table 1:** Description of the studied dog population

Group of dogs	Number of dogs	Mean age (month ± SD)	Min–Max (months)	Mean body weight (kg ± SD)	Min–Max (kg)
1.	19	9.21 ± 3.04	2–12	23.49 ± 6.05	13.0–34.0
2.	19	25.31 ± 7.13	13–36	31.52 ± 4.47	26.0–40.0
3.	19	51.63 ± 8.05	37–60	33.91 ± 4.87	25.0–42.70
4.	19	94.52 ± 20.51	61–144	32.69 ± 4.16	23.0–38.0
					
Males	37	49.11 ± 29.36	7–108	32.18 ± 7.52	13.0–42.70
Females	39	36.56 ± 31.45	2–144	28.72 ± 4.46	15.40–35.0
					
All dogs	76	42.67 ± 30.90	2–144	30.40 ± 6.35	13.0–42.70

**Table 2:** Measured concentrations of the urine biomarkers

Group of dogs	Mean CTX-I (ng/ml ± SD)	Min–Max CTX-I	Upper 95% for CTX-I	Mean CTX-II (ng/ml ± SD)	Min–Max CTX-II	Upper 95% for CTX-II	Mean TIMP-I (ng/ml ± SD)	Min–Max TIMP-I	Upper 95% for TIMP-I
1.	3.26 ± 1.17	1.34–5.22	–	1 868.45 ± 964.98	169.62–3 970.71	–	396.75 ± 188.64	43.20–669.21	–
2.	3.63 ± 0.97	1.91–5.51	–	1 959.07 ± 640.91	265.69–3 393.55	–	431.69 ± 140.72	160.99–642.63	–
3.	3.20 ± 0.96	0.87–4.46	–	2 132.03 ± 566.58	819.47–2 982.10	–	404.83 ± 145.98	84.07–619.66	–
4.	3.05 ± 1.48	0.56–5.82	–	2 016.27 ± 902.37	447.26–3 627.55	–	337.93 ± 160.52	92.25–600.67	–
									
Males	3.32 ± 1.13	0.83–5.51	5.05	1 906.01 ± 899.07	169.62–3 393.56	3 196.73	381.88 ± 144.95	84.07–602.302	586.60
Females	3.25 ± 1.20	0.56–5.82	4.99	2 077.39 ± 641.27	585.38–3 970.71	3 046.65	403.15 ± 175.35	43.20–669.21	640.97
									
All dogs	3.29 ± 1.16	0.56–5.82	5.05	1 993.95 ± 777.04	169.62–3 970.71	3 204.26	392.80 ± 160.56	43.20–669.21	606.64

CTX-I C = C-terminal cross-linked telopeptide type I collagen; CTX-II C = C-terminal cross-linked telopeptide type II collagen; TIMP-I = tissue inhibitor of metalloproteinase I

**Table 3:** Measured concentrations of the blood biomarkers

Group of dogs	Mean MMP-9 (ng/ml ± SD)	Min–Max MMP-9	Upper 95% for MMP-9	Mean PIICP (ng/ml ± SD)	Min–Max PIICP	Upper 95% for PIICP
1.	81.81 ± 38.43	16.17–188.04	–	23.20 ± 8.86	7.27–42.15	–
2.	80.82 ± 30.44	24.84–114.99	–	17.79 ± 3.37	11.74–24.29	–
3.	109.93 ± 78.96	10.41–327.87	–	16.34 ± 8.08	3.35–35.54	–
4.	86.85 ± 35.91	36.71–187.89	–	19.44 ± 6.41	5.61–31.13	–
						
Males	98.64 ± 61.40	10.41–327.87	206.25	18.57 ± 7.01	5.23–35.54	31.02
Females	81.52 ± 35.45	16.17–188.04	132.45	19.79 ± 7.67	3.35–42.15	33.52
						
All dogs	89.85 ± 50.21	10.41–327.87	187.93	19.19 ± 7.33	3.35–42.15	31.71

MMP-9 = matrix metalloproteinase 9; PIICP = Procollagen II Carboxy Terminal Propeptide

The mean age in the first group, represented by the 2 to 12 months old dogs, was 9.21 ± 3.04 months old, in the second group (13–36 months) it was 25.31 ± 7.13 months old, in the third (37–60 months) group, the mean age was 51.63 ± 8.05 months old, and, in the fourth group (more than 61 months), it was 94.52 ± 20.51 months old. The average body weight in each group was as follows: 1^st^ group was 23.49 ± 6.05 kg, 2^nd^ group was 31.52 ± 4.47 kg, 3^rd^ group was 33.91 ± 4.87 kg, and 4^th^ group was 32.69 ± 4.16 kg.

Statistically, the body weight was not different between the groups, only the first group with the youngest dogs had a lower weight compared to the other groups (*P* < 0.05). The weight of all females was statistically insignificantly lower than that of males, and the age of females was, on the contrary, slightly higher.

The mean values of the biomarkers ± SD were 3.29 ± 1.16 ng/ml for the urine biomarker CTX-I, 1 993.95 ± 777.04 ng/ml for CTX-II, and 392.80 ± 160.56 ng/ml for TIMP-I. Based on the obtained values of the mean 95%, we expect a standard of 5.05 ng/ml for CTX-I, 3 204.26 ng/ml for CTX-II, and 606.64 ng/ml for TIMP-I. The differences between the females and males were non-significant.

TIMP-I increased with the increasing age up to the second group, after which there was a decrease from the third group. CTX-II had a similar trend, but the peak occurred only in the third group, from which it then decreased. CTX-I gradually decreased with the increasing age of the dogs.

The mean values of the biomarkers ± SD were 89.85 ± 50.21 ng/ml for the blood biomarker MMP-9 and 19.19 ± 7.33 ng/ml for PIICP. Based on the obtained values of the mean 95%, we expect a standard for of 187.93 ng/ml for MMP-9 and 31.71 ng/ml for PIICP. The differences between the females and males were non-significant.

PIICP decreased with the increasing age, but, in the last, oldest group, we noted a slight increase. We recorded the peak of MMP-9 in the third group, while the value was almost the same in the other groups.

## DISCUSSION

MMPs are important for normal bone development and bone remodelling after trauma. The experimental deletion of MMPs in mice, particularly MMP-9, leads to impaired bone development (Davies et al. 2017).

In the pathophysiology of osteoarthritis, collagenases trigger the degradation of type II collagen, after which the resulting fragments become susceptible to degradation by other enzymes, such as gelatinase MMP-9. One of the physiological roles of MMPs is the splitting and building of connective tissues, for example, collagen and elastin, components of the extracellular matrix that provide structural support to cells. The dysregulation of MMPs or their inhibitors may be a key event in the pathophysiology of osteoarthritis (Alilovic et al. 2023).

The increased breakdown of the extracellular cartilaginous matrix is catalysed by matrix metalloproteases (MMPs) and aggrecanases (disintegrin and metalloproteinase with thrombospondin motifs). The MMP activity is strictly regulated by the endogenous tissue inhibitors of metalloproteases (TIMPs), which form inhibitory complexes with MMPs in a ratio of 1 : 1 (Hegemann et al. 2002). The endochondral activity of metalloenzymes can be disturbed by inappropriate mineral nutrition, which increases the likelihood of skeletal diseases.

In a study by Hegemann et al. (2002), it was quoted that not the serum, but the synovial biomarkers can serve as a predictive factor of stifle joint synovitis, the methodological statement in the study does not inform the reader about the age of studied animals and it also does not objectively exclude other pathologies in the studied animals, for example, oncological diseases in the control group of euthanised dogs.

In a study by Fujita, the MMP family consists of zinc endopeptidases that are structurally and functionally related and play a major role in the processes of OA and rheumatoid arthritis (Fujita et al. 2005).

In a recent study (Alilovic et al. 2023), Tornjak dogs with radiographic CHD had significantly lower serum concentrations of HA and higher concentrations of PIICP and MMP9 activity compared to dogs with radiographically normal hips (*P* < 0.05) (Alilovic et al. 2023).

The data itself from different literary sources varies and even our groups did not offer consistent results, but we contributed by describing the values of one picked breed (German Shepherd dog).

C-terminal telopeptide of type I collagen (CTX) is considered as a bone resorption marker. Collagen type I is synthesised in the bone and degraded to small peptide fragments that are released into the blood (Tymczyna et al. 2012). Changes in the bone turnover can be detected in as early as 3 months by measuring the CTX concentrations, whereas 12 to 24 months may be required to detect any changes in the bone density by radiographic methods. In addition, testing for CTX is non-invasive and can be repeated often. The CTX test is most useful for monitoring the response to antiresorptive therapy (Nikahval et al. 2016).

The degradation products of type II collagen have been studied in numerous studies, among them, C-telopeptide of type II collagen (CTX-II) is one of the most studied markers. Elevated levels were demonstrated in patients with OA compared with asymptomatic subjects or those without radiographic signs of OA. A more recent study showed the predictive value of urinary levels of CTX-II for the loss of cartilage assessed by magnetic resonance imaging (MRI) (Duclos et al. 2010).

The physiological values have not yet been described in veterinary medicine and therefore we focused on determining the physiological value of one type of breed, considering that this breed is most often represented in our clinic and is also characterised by frequent diseases of the skeletal system and high physical activity. So, we had all three aspects mentioned above summarised in one type of dog breed.

In human medicine, circulating metalloproteinases (MMPs) and their inhibitors TIMP are indices of the vascular and cardiac tissue matrix turnover. However, the temporal changes and relationship of these parameters to the age, sex, ethnicity and exercise have been poorly defined, even in healthy individuals. Therefore, they were studied, especially the plasma levels of MMP-2 and -9 and TIMP-1 and -2, as the major metalloproteinases (and their inhibitors) implicated in pathophysiology of vascular disease in humans (Tayebjee et al. 2004).

The age and exercise both have modest effects on the circulating concentrations of TIMP-1 and-2. MMP-9 appears lower in individuals of Far Eastern/Chinese origin regardless of the age or sex (Tayebjee et al. 2004).

Although urinary measurements of collagen degradation provide valid estimates of bone resorption, their clinical application is hampered by pronounced analytical and biological variability. Therefore, immunoassays for the determination of such parameters in the serum have been developed (Woitge et al. 1999).

Changes in urinary and serum markers were similar in most metabolic bone diseases. However, differentiation between healthy controls and primary vertebral osteoporosis, or primary hyperparathyroidism, was improved by the serum markers. In multiple myeloma, all serum and urinary markers were elevated. In breast cancer with bone metastases, skeletal involvement was reflected by significant increments. In patients with hypercalcemia of malignancy before and after treatment with pamidronate, pamidronate-induced changes in biomarkers were most pronounced for CTX (Woitge et al. 1999). Hepatic dysfunction, renal failure were associated with elevated levels of all serum markers. In conclusion, measurements in serum reflect bone resorption to the same extent as the urinary indices. Since serum markers circumvent some of the limitations of urinary measurements, their use potentially improves the assessment of skeletal disorders (Woitge et al. 1999). Finally, we would like to mention some limitations of our study. The first is that non-validated ELISA kits were used for the analysis, so it is necessary to approach the results with caution. The number of animals in the study is not sufficient to establish reference values in the usual way, however, it is large enough to assess the physiological fluctuation in the mentioned groups. Urinary biomarker concentrations can be significantly influenced by the urine density in dogs, but their determination from a 24-hour urine sample is not standardised in human medicine studies either. Despite these limitations, we believe that our study will be beneficial in the field of markers of cartilage damage in dogs.

In conclusion it could be stated, that numerous studies have shown the importance of breed-related differences between the haematological and biochemical results in veterinary medicine. It might be possible that the values of the biomarkers are also related to the respective breed or any age category, thus further studies are necessary to accurately determine the reference values and verify the specificity of the levels for individual diseases in dogs of different breeds.
